# Efficacy of Pembrolizumab as Conversion Therapy in Deficient Mismatch Repair/Microsatellite Instability-High Metastatic Liver Colorectal Cancer Associated With Lynch Syndrome: A Case-Based Analysis

**DOI:** 10.7759/cureus.97493

**Published:** 2025-11-22

**Authors:** Andrés de Jesús León-Sandí, Jimena Agüero-Moraga, Allan Ramos-Esquivel

**Affiliations:** 1 Oncology, School of Medicine, University of Costa Rica, San José, CRI; 2 School of Medicine, University of Costa Rica, San José, CRI

**Keywords:** colorectal cancer, immunotherapy, lynch syndrome, metastasis, pembrolizumab.

## Abstract

Immunotherapy has emerged as a new standard of care for patients with unresectable or metastatic colorectal cancer characterized by microsatellite instability-high (MSI-H) or deficient mismatch repair (dMMR), owing to its superiority compared with conventional chemotherapy, as demonstrated in recent clinical trials. However, to date, no clinical trial has evaluated the role of immunotherapy as a neoadjuvant strategy aimed at converting potentially resectable hepatic metastases into candidates for curative surgery. In this report, we present the case of a patient with Lynch syndrome who developed hepatic metastasis after a right hemicolectomy for colon adenocarcinoma. The patient was treated with upfront pembrolizumab as conversion therapy following discussion in a multidisciplinary tumor board. After six cycles of immunotherapy, the patient had stable disease according to the Immune Response Evaluation Criteria in Solid Tumors (iRECIST), and a pathological complete response was achieved following complete resection of the liver metastasis.

## Introduction

Colorectal cancer (CRC) represents the third most frequently diagnosed malignancy worldwide, with rising incidence rates in developing countries and among younger populations [1]. Several pathophysiological mechanisms have been proposed to explain its carcinogenesis. Among these, microsatellite instability-high (MSI-H) is a key molecular feature observed in approximately 5-30% of CRC cases and carries both prognostic and predictive implications [2,3]. This phenotype results from a deficient DNA mismatch repair (MMR) system, caused by inherited or acquired loss of expression of one or more of four critical genes: *MLH1*, *PMS2*, *MSH2*, and *MSH6* [1]. Deficient expression of any of these genes leads to a dysfunctional MMR complex, allowing the accumulation of unrepaired DNA replication errors and the development of MSI-H, which refers to variations in the length of repetitive DNA sequences compared to germline DNA [3,4]. Lynch syndrome (LS) is a hereditary, autosomal-dominant disorder caused by germline mutations in MMR genes and is strongly associated with the MSI-H phenotype, accounting for approximately 2-4% of all CRC cases [1,2]. Although LS involves inherited monoallelic mutations, biallelic inactivation is required for MMR deficiency to manifest [3].

Pembrolizumab, an IgG4 monoclonal antibody, binds to programmed cell death protein 1 (PD-1), thereby preventing its interaction with its ligands programmed death-ligand 1 and 2 (PD-L1, PD-L2) and ultimately inhibiting PD-1-mediated suppression of T-cell activity [1,4]. The US Food and Drug Administration (FDA) approved pembrolizumab for use in patients with unresectable or metastatic CRC (mCRC) exhibiting MSI-H or deficient mismatch repair (dMMR) [4]. However, current clinical guidelines do not recommend the use of immunotherapy (IO) in patients with potentially resectable liver metastases (conversion therapy to enable surgical resection), mainly because no phase III clinical trials have specifically evaluated its role in patients with borderline resectable colorectal liver metastases [5].

In patients with CRC involving lymph nodes (stage III) or distant organs (stage IV), IO has demonstrated improved outcomes compared with chemotherapy [6-12]. IO exerts its effect by modulating the immune system to target tumor cells, primarily through regulation of the tumor microenvironment to enable an effective immune response [4].

In this report, we present the clinical outcomes of a patient with LS treated with pembrolizumab as upfront therapy for borderline resectable colorectal metastasis, with the aim of supporting the role of IO as conversion therapy.

## Case presentation

Initial diagnosis

A 45-year-old woman with no other significant medical history was diagnosed at the age of 41 with right-sided colon cancer after presenting with abdominal pain and mild iron-deficiency anemia. The carcinoembryonic antigen (CEA) level was measured at 1.67 ng/mL (range: 0-5 ng/mL) with no evidence of metastatic spread at diagnosis according to computed tomography (CT) scans of that time.

Her maternal family history was remarkable for CRC. Further investigation, in collaboration with genetic counseling, revealed that one maternal aunt had developed three metachronous colon cancers at the ages of 39, 42, and 49. Another maternal aunt died at age 35 from colon cancer. The maternal grandmother had colon cancer at 40. Additionally, two of the grandmother’s brothers were also diagnosed with colon cancer, and one of her nephews was diagnosed with pancreatic cancer at age 40. Family history from the paternal side was unknown. Based on this information, the family met Amsterdam I and Bethesda criteria, strongly suggesting LS, likely due to an *MSH2* mutation.

In December 2021, after surgical removal of the right colon, the pathological stage was pT3 pN0 (0/22) M0 (Stage II). The patient was not considered a candidate for adjuvant chemotherapy due to the absence of high-risk features and because of the presence of immunohistochemical analysis with loss of expression of MSH2 and MSH6, and preserved expression of PMS2. Cytoplasmic immunohistochemistry for *BRAF* V600E was negative. Molecular testing detected a germline pathogenic variant in the *MSH2* gene: a heterozygous deletion in exon 7, c.1077_1276del200, consistent with LS.

Recurrence and treatment

**Figure 1:**
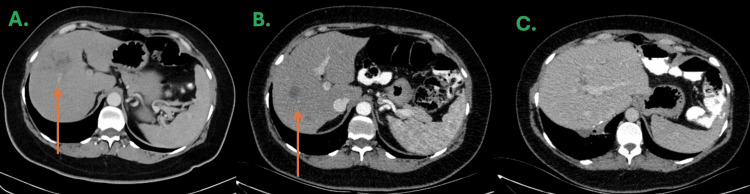
CT scans at baseline (A), after 10 cycles of pembrolizumab (B), and one year post–right hepatectomy (C), showing sustained absence of disease recurrence.

After 21 months of follow-up, in September 2023, a borderline resectable hepatic recurrence was identified by CT imaging involving segments IV and VI of the liver (Figure 1A). Triphasic CT scan revealed a focal area with a heterogeneous pseudonodular appearance that remains hypodense throughout the different phases of the triphasic study, possibly corresponding to necrosis, measuring approximately 37×28x20 mm with mild contrast enhancement and mild dilatation of the adjacent intrahepatic biliary ducts. 

Based on clinical assessment and tumor-board discussion, a treatment plan was recommended, including conversion therapy, followed by portal embolization, and subsequently an extended right hepatectomy. Given the high response rate of MSI-positive tumors to IO, treatment with pembrolizumab 200 mg IV every 21 days was initiated in October 2023. In November 2023, a portal vein embolization was performed to induce hypertrophy of the liver remnant. The patient experienced grade 2 hypothyroidism treated with levothyroxine after cycle 2, and grade 2 pneumonitis after the third cycle, requiring temporary suspension of pembrolizumab for one cycle and a short course of 10 days of oral prednisone (1 mg/kg) with complete resolution of symptoms. 

The preoperative CT scan described an ill-defined hypodense lesion without contrast enhancement located between hepatic segments IV and VI, measuring approximately 20×19×23 mm. No significant changes were noted compared to prior imaging, corresponding to partial response according to Immune Response Evaluation Criteria in Solid Tumors (iRECIST) (Figure 1B).

Surgery and pathology

In April 2024, 15 days after the tenth three-weekly pembrolizumab cycle, a right hepatectomy was performed. Intraoperatively, a lesion was observed involving segments V, VI, and VII. Intraoperative ultrasound did not reveal any other suspicious lesion. The hepatoduodenal ligament was dissected, and both the right hepatic artery and right portal vein were identified and ligated. The right hepatectomy was completed using vascular stapling equipment. 

Histopathological examination revealed no evidence of carcinoma, consistent with a complete pathological response, and clear resection margins. There was extensive, multifocal, globular deposition of homogeneous foreign material consistent with prior use of a thrombotic agent for embolization. The patient completed one year of treatment with pembrolizumab after surgery. 

Subsequently, in September 2024, the CT scan revealed only a fluid collection around the surgical site, with no evidence of active disease.

Follow-up

The patient was monitored according to clinical guidelines [5], with physical examinations and serum CEA measurements performed every three to four months, and CT scans obtained every six months. A colonoscopy conducted one year after her last surgical procedure showed no evidence of tumor recurrence. As of September 2025, the patient remained disease-free and exhibited no long-term toxicities related to her therapy. The most recent CT scan demonstrated postoperative absence of right hepatic segments (VI and VII) with fibrotic bands extending toward the diaphragm and no focal lesions identified (Figure 1C). Due to the diagnosis of LS, the patient will undergo lifelong surveillance.

## Discussion

This case study adds valuable “real-world” evidence for successful outcomes after conversion therapy with pembrolizumab in a patient with borderline resectable liver metastasis for CRC. Of note, despite the presence of a partial response per iRECIST criteria, pathological examination revealed a complete response (CR) with the disappearance of the previous lesion after 10 cycles of IO. This finding is noteworthy since no clinical trials to date have explored the efficacy and safety of IO as conversion therapy for patients with MSI/dMMR metastatic colon cancer. 

Burden of mCRC

The burden of mCRC should be highlighted due to its relatively high prevalence and because approximately one-quarter of patients with this neoplasia present with or develop metastatic disease, with the liver being the most common site of metastasis, occurring in approximately 70% of cases [13]. For many of these patients, despite the spreadable disease, there is a chance of cure after complete resection of liver metastases, after achieving R0 resection (leaving no tumor at the margin). Indeed, some series have reported five-year survival rates of 20%-45% for those patients achieving R0 resections after liver metastasectomy [14]. For that reason, multidisciplinary tumor board discussion is highly recommended to determine the feasibility of upfront surgery or the use of systemic treatment to convert borderline resectable disease to resectable. 

Rationale for conversion therapy

Although surgical resection remains the standard of care, and in select cases may be performed without preoperative chemotherapy, the combination of surgery and systemic chemotherapy has been shown to reduce the risk of recurrence. Specifically, this combined approach has demonstrated a 25% reduction in the risk of disease progression at three years compared to surgery alone in patients with resectable liver metastases [15]. However, with chemotherapy alone, only 15% of patients with initially unresectable tumors become candidates for surgical resection [16]. Further studies support the role of highly active and moderately toxic regimens such as 5-fluorouracil, leucovorin, oxaliplatin, and irinotecan (FOLFOXIRI) plus bevacizumab to improve the chance of complete resection (R0) in comparison to other regimens with no use of anti-angiogenics [17]. Current clinical guidelines suggest that the addition of a targeted agent to a cytotoxic doublet or triplet is the most effective treatment in potentially resectable mCRC. 

Role of IO in MSI-H disease

Despite these recommendations, emerging evidence indicates that MSI-H/dMMR tumors are less responsive to conventional chemotherapeutic agents [4]. For instance, Shulman et al. reported inferior overall response rates in MSI-H mCRC compared to microsatellite-stable (MSS) disease under standard regimens such as FOLFOX and FOLFIRI [18]. In contrast, recent data from the KEYNOTE-177 trial demonstrated that pembrolizumab significantly improved progression-free survival (PFS), objective response rate (ORR), and CR compared with standard chemotherapy in patients with dMMR/MSI-H mCRC. Notably, the ORR increased from 33.1% to 43.8%, while the CR rate rose from 3.9% to 11.1% [8]. These effects are likely attributable to the inherent immunogenicity of dMMR/MSI-H tumors, which possess a high mutational burden leading to increased neoantigen presentation and a highly inflamed tumor microenvironment rich in immune cell infiltration [4]. In line with these findings, the KEYNOTE-164 [19] and KEYNOTE-016 [20] trials consistently demonstrated that pembrolizumab achieved ORRs ranging from 33% to 56%, with CRs in up to 29% of patients.

The complete pathological response observed in this case is consistent with recent findings from clinical trials evaluating IO in CRC. For instance, the CheckMate 8HW trial reported that patients with MSI-H/dMMR CRC treated with dual immune checkpoint blockade (nivolumab plus ipilimumab) achieved a significant improvement in PFS compared with conventional chemotherapy, along with an ORR of 70% [9]. Similarly, the NICHE-2 and NICHE-3 trials demonstrated unprecedented pathological CR rates (68%) in patients with MSI-H operable colon cancer treated with nivolumab plus ipilimumab or nivolumab plus relatlimab, respectively [11,12]. Likewise, single-agent dostarlimab administered for six months achieved a 100% clinical CR rate in patients with MMR-deficient stage II or III rectal cancer [10].

Despite these encouraging results, no randomized clinical trial has yet demonstrated the superiority of IO over conventional chemotherapy and biologic agents in improving R0 resection rates in liver mCRC [5]. We hypothesize that, given the high response rates of IO in MSI-H/dMMR mCRC, single-agent or dual-agent IO could become the standard of care for potentially resectable disease in this population, as recent clinical trials have shown in an MSI-H/dMMR setting. Besides, previous studies have suggested a possible relationship between tumor burden and pathological response (as observed in NICHE-2) [11]. Of particular interest is the duration of preoperative IO, as trials have indicated that pathological response rates depend on treatment length. For example, the NICHE-3 trial allowed eight weeks from initiation to surgery, compared with six weeks in NICHE-2, while the NCT0416577 trial permitted at least nine cycles of dostarlimab in locally advanced rectal cancer to assess clinical response [11-13]. These variations may reflect a direct correlation between treatment duration and pathological CR rate. Additional questions remain regarding optimal IO dose, the best checkpoint target (PD-1 vs PD-L1), and whether monotherapy or combination strategies yield superior outcomes. Moreover, imaging-based response assessment warrants reevaluation, as prior studies have shown that iRECIST criteria do not consistently correlate with pathological CR [4].

Safety considerations

Although no serious treatment-related adverse events occurred in this case, it is important to note that in the KEYNOTE-177 trial, grade ≥3 adverse events were reported in 22% of patients receiving pembrolizumab [3,4]. Common adverse effects included diarrhea, fatigue, nausea, abdominal pain, and decreased appetite. Immune-related events of special interest included hypothyroidism, hyperthyroidism, colitis, pneumonitis, adrenal insufficiency, and hepatitis [4]. Prompt recognition of such side effects is crucial to avoid clinical deterioration, as illustrated in our patient, whose respiratory symptoms required immediate evaluation and management of potential pneumonitis. Despite these potential side effects, achieving a durable CR, avoiding chemotherapy-related toxicity, and obtaining a successful surgical outcome are potential advantages of using IO in a conversion setting before liver metastasectomy.

## Conclusions

In conclusion, this case highlights a potential paradigm shift in offering conventional chemotherapy and targeted agents to patients with potentially resectable liver mCRC. In this patient, pembrolizumab allowed us to avoid the hepatic toxicity associated with oxaliplatin and irinotecan, while achieving a pathological CR following R0 resection. Timely identification of the MSI-H/dMMR profile is essential to guide effective and personalized treatment, underscoring the fundamental role of IO as a safe and effective therapeutic alternative for mCRC associated with microsatellite instability, even in the setting of potentially resectable liver disease. Although the single-case nature of our report limits its generalizability, further studies are needed to evaluate this approach. Future clinical trials should explore key questions such as optimal treatment duration, timing of surgery, and management of potential adverse effects before changing current clinical practice.
